# Groundwater arsenic and education attainment in Bangladesh

**DOI:** 10.1186/s41043-015-0029-6

**Published:** 2015-10-26

**Authors:** Michael P. Murray, Raisa Sharmin

**Affiliations:** 1 Department of Economics, Bates College, Lewiston, ME 04240 USA; 2 University of Waterloo, Waterloo, Canada

**Keywords:** Arsenic, Education, Groundwater, Bangladesh

## Abstract

**Background:**

Thousands of groundwater tube wells serving millions of Bangladeshis are arsenic contaminated. This study investigates the effect of these wells on the education attainment and school attendance of youths who rely on those wells for drinking water.

**Methods:**

The analysis combines data from the 2006 Bangladesh Multiple Indicator Cluster Survey (2006 MICS) and the National Hydrochemical Survey (NHS) of Bangladeshi tube wells’ contamination conducted between 1998 and 2000. The study uses multiple regression analysis to estimate the differences in education attainment and school attendance among the following: (i) youths who live where tube wells are safe, (ii) youths who live where tube wells are unsafe but who report drinking from an arsenic-free source, and (iii) youths who live where tube wells are unsafe but who do not report drinking from an arsenic-free source.

**Results:**

Controlling for other determinants of education attainment and school attendance, young Bangladeshi males who live where tube wells are unsafe (by Bangladeshis standards) but who report drinking from arsenic-free sources are found to have the same education attainment (among 19- to 21-year-olds) and school attendance (among 6- to 10-year-olds), on average, as corresponding young Bangladeshi males who live where wells are safe. But young Bangladeshi males who live where tube wells are unsafe and who do not report drinking from an arsenic-free source attain, on average, a half-year less education (among 19- to 21-year-olds) and attend school, on average, five to seven fewer days a year (among 6- to 10-year-olds) than do other Bagladeshi males of those ages. The estimated effects for females are of the same sign but much smaller in magnitude.

**Conclusion:**

Bangladeshi public health measures to shift drinking from unsafe to safe wells not only advance good health but also increase males’ education attainment.

## Background

While groundwater arsenic has plagued many countries, including Argentina, Mexico, India, Nepal, the USA, and Vietnam [1], in Bangladesh, the problem has been called “the largest poisoning of a population in history” [2–4].

Arsenic poisoning is calamitous. The World Health Organization (WHO) reports that drinking arsenic-contaminated water on a regular basis increases the risk of numerous cancers and can lead to skin pigmentation changes and hyperkeratosis [5]. Drinking from arsenic-contaminated tube wells has chronically poisoned millions of Bangladeshis; building on Sohel et al. [6] and Flanagan et al. [7] estimated in 2012 that in Bangladesh over 40.000 deaths per year are due to arsenic poisoning. According to Chen et al. [8], arsenic-contaminated drinking water more than doubled Bangladeshi’s lifetime mortality risk from cancers of the liver, bladder, and lung (229.6 vs 103.5 per 100,000 population).

Extensive reviews are available for studies of the physical, neurological, social, and psychological consequences of arsenicosis in Bangladesh (e.g., [1, 9, 10]). The literature, however, appears mute on arsenic’s effects on education attainment. Education generally increases lifetime earnings, with primary school education having the largest return [11, 12]. Moreover, greater education attainment by a country’s population has been found to spur economic growth [13–15]. Given the economic importance of education, it seems worth asking whether the physiological effects and social stigma associated with drinking arsenic-contaminated water reduce education attainment for those who grow up drinking such water. In this paper, we report our estimates of the effects of drinking arsenic-contaminated water on both primary school attendance by young Bangladeshi boys and total years of education completed by young Bangladeshi males.

Children are struck especially hard by arsenic poisoning. Given this study’s focus on school attendance and education attainment, neurological and social effects of arsenicosis are particularly pertinent as reduced cognitive capacity inhibits education attainment, and shunning by peers could discourage school attendance.

Studies have established adverse effects of arsenic on children’s verbal comprehension, long-term memory [16], attention [16], cognitive development [17, 18], neurobehavioral development such as pattern memory and switching attention [19], and intelligence [20, 22]. Asadullah and Chaudhury [17] find significant effects of arsenic on mathematics scores for Bangladeshi children. According to Chowdhury et al. [23] and Khandoker et al. [24], victims of lesions and blemishes frequently experience being ostracized and shunned within their families and local communities, even to the extent of being excluded from marriage. Nasreen [25] provides several case studies of the adverse social effects of arsenicosis in Bangladesh.

Millions of groundwater wells were installed in Bangladesh from the 1970s onward [8], for the most part with funds from international agencies, with the goal of ending reliance on unsanitary surface water for drinking [26]. That a million or more of these wells were arsenic contaminated was long unknown [2–4, 27]. Estimates in the late 1990s [28, 29] revealed that some 35 million Bangladeshis were drinking their water from seriously contaminated wells. Subsequent efforts by the Bangladeshi government to alert households to the threat of arsenic-contaminated tube wells has reduced the proportion of the population drinking from such wells, but the number has remained high—the Bangladesh Bureau of Statistics and the United Nations Children’s Fund [30] have estimated that 12.5 % of Bangladeshis, or 20 million people, still regularly drank arsenic-contaminated water in 2012–2013. An extensive literature assessing efforts to reduce arsenic poisoning has developed (e.g., [19] [31–34]).

The progress made by Bangladesh in reducing households’ reliance on arsenic-contaminated wells is evident in Table 1 which presents Bangladeshis’ exposure to various levels of arsenic in their drinking water in 2000, 2009, and 2012–2013. In 2000, 42.1 % of wells had above the 10 parts per billion (ppb) arsenic that the World Health Organization deems the upper limit for safe drinking water [35]. Of wells, 24.9 % were above the Bangladesh safety limit of 50 ppb [17]. By 2012–2013, 24.8 % of households were found to be drinking water above the 10-ppb limit and 12.5 % above the 50-ppb limit.^1^ Argos et al. [36] estimate that drinking groundwater containing more than 150 ppb of arsenic causes almost a doubling of mortalities from all causes. Flanagan et al. [8] estimate that the percentage of households exposed to such a high level of arsenic was 8.9 % in 2000 and 4.8 % in 2009.

**Table 1:** Drinking water arsenic contamination exposure

Arsenic level (ppb)	Wells % above 2000^a^	Households % above level^b^ 2009	Households % above level^c^ 2012	Male youths % above level^d^ 2006
10	48	32	24.8	62
50	25	13.4	12.5	34
100	16	6.2	–	18
200	9	3.4	2.8	8
300	5.1	1.8	–	2

*ppb* parts per billion

^a^Source: [27], Table 6.7

^b^Source: [7], Table 1

^c^Source: [29], Table WQ.2

^d^Authors’ calculations from 2006 MICS and NHS data

There is a regional variation in Bangladesh’s groundwater arsenic levels. Contamination is greatest in the south and southeast of the country and least in the northwest and in north-central Bangladesh [28]. However, highly contaminated groundwater has been found in some locales in the generally low-arsenic regions of northern Bangladesh [28]. In 2009 and 2012–2013, contamination rates in rural areas were about double than those in cities [30, 37].

## Methods

### Data and sampling

We relied on two publicly available data sets: (i) the 2006 Bangladesh Multiple Indicator Cluster Survey (2006 MICS) that was carried out by the Bangladesh Bureau of Statistics and UNICEF [37] and which has been cited as an example of “good practice” for international data collection [38], and (ii) the oft-cited National Hydrochemical Survey (NHS) of wells conducted between 1998 and 2000 by the Department of Public Health Engineering of Bangladesh in consultation with the British Geological Survey [28].

The 2006 MICS is a nationally representative, randomly sampled, household survey with a response rate of 92.5 % [37]. The survey provides data on household socio-economic variables, including the head’s and each individual’s education attainment, local environment questions such as proximity of the household to industrial pollution sources, and questions specifically about local well-water arsenic contamination.

The NHS provided chemical test results for 3534 boreholes from 61 of Bangladesh’s 64 districts. The goal of the NHS was a random sample of tube wells, but logistical problems barred fully realizing this ideal [28]. The NHS reports surveyed well locations at the village level. We aggregated the arsenic levels to the sub-district level, the finest geographic detail available in the 2006 MICS data. Sub-districts in Bangladesh average about 150 km^2^., or 60 mi^2^. Districts average 2300 km^2^ or 890 mi^2^. We designated as ‘unsafe’ sub-districts with average NHS well-water arsenic levels above Bangladesh’s 50 ppb standard; we designated all other districts ‘safe’.

Choosing a sub-sample of individuals to study for arsenic’s effects on years of education required judgment. Tube wells were far from ubiquitous in Bangladesh in the late 1970s and early 1980s. Schoenfeld [33] found some urban areas in which under 40 % of households used tube wells in 1977, and clinical reports of physical effects did not begin until the early 1980s [2, 3, 27]. Thus, we did not know exactly which birth cohorts were fully exposed to the groundwater arsenic levels measured in the NHS. Estimates of arsenic’s effects based upon individuals born before the general use of tube wells would underestimate those effects, but estimates based upon individuals too young would miss the full effect of arsenic on education. We estimated, by sex, well-water arsenic’s effects on education attainment for youths who were aged 19 through 21 when sampled for the 2006 MICS, i.e., individuals born between 1985 and 1987. Of these ages, there were 7451 males and 9270 females in the 2006 MICS; one seventh of these youths were in sub-districts for which we had no tube well data and were therefore omitted from our analysis.

We chose individuals born between 1985 and 1987 for two reasons. First, the first clinical reports of arsenic poisoning occurred about this time [2, 27]; older individuals might have not felt the full impact of the growing number of arsenic-contaminated tube wells. Second, only 20 % of the sampled individuals between 19 and 21 years of age reported being in school and less than 7 % of the 22-year-olds reported being in school, so by age 19, most of arsenic’s adverse effects on education had occurred.

Matching young men and women to the wells they drank from since childhood is not exact since where they lived in 2006 might not be where they lived for most of their childhoods. This source of measurement error looms largest for young adults who have moved from their parents’ home because such individuals might well have moved away from their childhood sub-district. To reduce the attenuation bias from such measurement problems, we restricted attention to 19–21-year-olds who still lived with their parents. In the 2006 MICS, 80 % of the 19–21-year-olds still lived with their parents.

The Bangladeshi school system required school enrollment through grade V, which corresponds to ages 6 through 10. We examined, by sex, whether drinking arsenic-contaminated water affected school attendance of children aged 6 to 10 in 2006 when they were sampled for the 2006 MICS.

### Key variables

This study focused attention on four variables: (i) an individual’s years of education attained, (ii) the number of school days a child attended in the past week, (iii) whether the sub-district in which an individual lived in 2005 had safe or unsafe groundwater, and (iv) whether an individual was reported by the survey respondent to drink from an arsenic-free source (which we shall refer to as ‘the individual drinks safely’). The NHS provided arsenic levels; the 2006 MICS provided the other three variables. The first two variables are integer valued; the second two are dummy variables.

Due to lack of extensive household-specific, year-by-year, data on the consumption of arsenic-contaminated water in Bangladesh, we of necessity measured exposure to arsenic-contaminated water by the average contamination level of local tube wells sampled in the 1998–2000 NHS. This 1998–2000 data was quite pertinent for several reasons. First, the natural hydrological traits of wells change little over time [39, 40]; we concluded from this that local tube well contamination was relatively stable from the mid- to late 1980s through 2006. Second, the 19–21-year-olds, whose total years of education by 2006 we studied, and the 6–10 year olds, whose school attendance we studied, grew up between 1985 and 2006, which were mostly years in which relatively few households were adjusting their behavior to avoid contaminated wells [33].

### Control variables

Drinking arsenic-contaminated water is certainly not the only determinant of education attainment. Our multiple regression analysis controls for the well-known major determinants of education attainment. Mare [41] established that in the USA, the number of one’s siblings, local-area economic conditions, and one’s parents’ financial resources and education are key determinants of a child’s education attainment. More recently, Li et al. [42] and Huang [43] have confirmed these findings in a developing country context. Holmes [44] further established the importance of local wage and employment opportunities for decisions about continued schooling. We constructed six groups of control variables from the 2006 MICS data specific to our concern about arsenic contamination and other potential health threats:

1. Contamination awareness and avoidance: survey respondents reported whether they had heard of the well-water arsenic problem and whether they drank from arsenic-free sources.
2. Wealth/income indicators: (a) the household’s *z*-score in the 2006 national distribution of wealth, (b) the square of the household’s wealth *z*-score, (c) the household head’s education level (seven categories), (d) the mean wealth *z*-score across sampled households in the sub-district in which the individual lives, and (e) the standard deviation of wealth *z*-scores for sampled households in the sub-district in which the individual lives. We view the mean and standard deviation of local wealth as indicating local income opportunities that compete with school for individuals’ time.
3. Family circumstances, including the education of the household head and the number of other children competing for the households’ resources.
4. Local environs indicators. Whether the sampled dwelling was as follows: (a) in a flood-prone area, (b) in a landslide-prone area, (c) located near industrial pollution, and (d) located near a garbage pile.
5. Housing security indicators: (a) did the respondent report security from eviction and (b) was the household squatting.
6. Indicator variables, one for each district, used to indicate where a household was located at the time of the survey.

We included in our regression models all the control variables in items 1–6, plus the individual’s age reported in the 2006 MICS. Separate measures of mother’s and father’s education are available in too few cases to support regression analysis; we instead use the education of the household head to capture parental education. We also do not have a count of the number of siblings a child has. To proxy for the number of siblings, we use for youths 19–21 the number of other youths in the household, and for children 6–10 the number of other children in the household 0–16 years of age.

In our regression analyses, approximately 10 % of cases from the 2006 MICS were lost due to missing values on control variables.

### Statistical analysis

We employed ordinary least squares (OLS) multiple regression analysis [45] to estimate the effects of tube wells’ arsenic contamination on school attendance of 13,556 six- to ten-year-olds and on the education attainment of 4511 nineteen- to twenty-one-year-olds. To infer causal relationships from observational data, as opposed to experimental data, suitable controls were required for potentially confounding variables. Multiple regression allowed inclusion of such controls in our analysis.

Multiple regression has long been used for analyzing education attainment, dating back to at least 1980 [41] and has more recently been employed for such studies in developing country contexts (e.g., [42–44]). For this paper, summary and regression statistics were all calculated using the regression routines of STATA 12™. The regression models all included dummy variables (binary zero-one variables) for the sub-district in which an individual lived in 2006. With many individuals in each sub-district, the regression disturbance terms were likely to be correlated within districts. The estimated standard errors we employed in the regressions accounted for this clustering of observations by sub-district [46].

## Results

Bangladeshis in the 2006 MICS had generally completed their educations by age 21; only 6.8 % of the sampled Bangladeshi 22-year-olds reported still being in school. The average years of education of the sampled 22-year-olds was 7.4 years, with little difference between males and females. In 2005, on average, sampled primary school children attended school 5.3 out of 6 days per week, again with little difference between males and females.

Table 1 reveals that young Bangladeshis in the 2006 MICS tended to live in areas with somewhat greater than average arsenic exposure, although somewhat less often in the most affected areas. In 2006, almost two thirds of the sampled Bangladeshis aged 21 or less lived in communities in which the average well’s arsenic level exceeded the WHO standard of 10 ppb or less [35]. Over a third of young Bangladeshis lived in communities in which the average well’s arsenic level exceeded Bangladesh’s own safe water standard of 50 ppb arsenic or less [20]; and 13 % lived in communities in which the average tube’s well-water contained 150 ppb arsenic or more, which is the level Argos et al. [36] associate with a near doubling of all-causes mortality. The third of the sampled young Bangladeshis living in sub-districts with on-average unsafe well-water were, on average, exposed to arsenic levels of 145 ppb in their sub-district’s well-water.

Authors’ computations from the 2006 MICS and the NHS reveal that youths in households with a head who had completed primary schooling were 1.1 % more likely to live in communities with on-average unsafe drinking water than household heads with less education. (We designated as “unsafe” sub-districts with average NHS well-water arsenic levels above Bangladesh’s 50 ppb standard; we designated all other districts “safe.”) Perhaps surprisingly, youths in households with a head who had greater than median wealth were 4.8 % more likely to be exposed to arsenic-contaminated water than youths whose household heads had less than median wealth. Given the large sample size of the 2006 MICS, these computed differences are statistically significant at all conventional significance levels. Given these differences in education and wealth, simple comparisons of the mean education attainment between youths in safe and unsafe drinking water communities will reflect not only the effects of arsenic but also the effects of differences in parental wealth and, to a lesser degree, education.

Awareness of the arsenic problem was quite high among households sampled in the 2006 MICS, as evidenced by Table 2. In general, both awareness of the arsenic problem and reported reliance on arsenic-free sources rose with the local level of arsenic in wells. (Again, all of the differences in the table are statistically significant.) This pattern is unsurprising since the government focused its policy efforts on the most at-risk areas, and, the greater the risk, the more reason for people to “spread the word” about the problem.

**Table 2:** Parental arsenic awareness and youths drinking safely

Percentage of youths with parents who in 2006 reported of having heard of arsenic problem	
Safe dub-districts^a^	72.7 %
Unsafe sub-districts^b^	92.9 %
Total	79.6 %
Percentage of youths in unsafe sub-districts who in 2006 were reported to drink safely	
Head did not complete primary school	50.2 %
Head completed primary school	66.6 %
Below-median wealth	47.0 %
Above-median wealth	65.5 %
Total	56.9 %

^a^Safe means tube well arsenic content in sub-district is ≤50 ppb

^b^Unsafe means tube well arsenic content in sub-district is >50 ppb

Table 2 also reveals that decisions to drink safely in unsafe sub-districts differed sharply with both parental education and wealth. Among sampled children living in sub-districts with unsafe water, those whose household heads had completed primary school drank safely 16.4 % more often than the sampled children whose household heads had less education, and those whose households had above-median wealth drank safely 15.5 % more often than the sampled children from less wealthy households. Simple comparisons of the mean education attainment between those who drank safely in the unsafe districts and those who did not reflect not just differences in arsenic consumption but also differences in parental education and wealth. To reliably estimate the causal effects of arsenic using the 2006 MICS data, one must control for parental wealth and education.

Table 3 summarizes the education attainment of the sampled Bangladeshi youths aged 19 to 21 and the attendance patterns for sampled Bangladeshi children age 6–10 in this nationally representative sample. Our interest is in whether drinking from arsenic-contaminated wells results in a lower number of years of education attained and/or in fewer days of school attended in the past week. We estimate the effect of drinking arsenic-contaminated water by comparing three groups of sampled individuals: (i) individuals who lived in sub-districts with safe groundwater, (ii) individuals who lived in sub-districts with unsafe groundwater who report drinking from arsenic-free sources, and (iii) individuals who lived in sub-districts with unsafe groundwater who did not report drinking from arsenic-free sources.

**Table 3:** Education attainment and school attendance in 2006 for young bangladeshis

	Males	Males	Females	Females
Age	Avg. years ed.	% in school	Avg. years educ.	% in school
15	5.96	53.2	6.70	56.2
16	6.48	45.3	7.16	45.2
17	6.96	42.0	7.40	32.2
18	6.87	29.4	7.35	23.2
19	7.51	23.3	7.49	17.7
20	7.19	22.0	7.38	12.7
21	7.91	24.3	7.39	11.7
	Boys 6–10		Girls 6–10	
Age	Avg. days attended last week		Avg. days attended last week	
6–10	5.26		5.32	
Mean education attainment in 2006 for youths 19–21				
Safe sub-districts	7.92 years			
Unsafe sub-districts	7.82 years			
Mean education attainment in 2006 for youths 19–21 in unsafe sub-districts				
Reported to drink safely	8.25 years			
Not reported to drink safely	7.18 years			

Source: Authors’ calculations from 2006 MICS

Education was compulsory for 6- to 10-year-olds; we focus on the school attendance of these children. For older youths, our concern is with their ultimate years of education attained. Since school participation rates were still nearly 30 % or higher for youths younger than 19, we choose to focus primarily on youths 19–21.

Table 3 summarizes the mean education attainment by the sampled 19–21-year-olds in sub-districts with safe water and in sub-districts with unsafe water. Table 3 also reports, for the sampled 19–21-year-olds who lived in the unsafe sub-districts, their mean education attainment was broken down by whether the youth drank safely or not. These data offer seemingly conflicting stories about how arsenic affects education attainment. Between safe and unsafe districts, the mean education attainment differed by 0.09 years. Since 40 % of the sampled youths in unsafe districts drank unsafely and 60 % drank safely, the 0.09 difference implies that those who drank unsafely averaged 0. 225 (i.e., 0.09/0.4) fewer years of education than others—groundwater arsenic seems to have mattered only modestly. But on average, the sampled youths in the unsafe sub-districts who drank safely attained 1.07 more years of education than their sampled neighbors who drank instead from contaminated sources—groundwater arsenic seems to have mattered quite a lot.

The seeming conundrum arises because differences in the mean education attainment reflect not only differing exposure to groundwater arsenic but also differing parental wealth and education (and differences in yet other determinants of education attainment, as well). To resolve the conundrum, we need to control for the multiple determinants of education attainment.

We report in Table 4 regressions of education outcomes on tube well contamination for the sampled males 19–21 years old. These regressions also included controls for parental wealth, parental education, and other likely determinants of education attainment. We do not report results for females because the estimated effects of living in an unsafe sub-district were much smaller in magnitude for females than for males. The smaller effects for females are consonant with the speculation of Asadullah [47] that observed lower levels of arsenic among Bangladeshi females [47, 48] are due to females drinking less per pound of body weight than do males (if this speculation is correct, a given level of a tube well’s toxicity is less consequential for local females than for local males).^2^

**Table 4:** Unsafe water’s effects on males’ years of education (19–21-year-olds) and days of school attended (6–10-year-olds)

Variable	Years of education		Days of school attended	
Regression	1	2	3	4
Unsafe water	−.501^a^ (0.134)	−.565^a^ (0.167)	−.119^b^ (0.053)	−.170^a^ (0.067)
Unsafe*drink safe	0.494^a^ (0.140)	0.497^a^ (0.141)	0.107^c^ (0.056)	0.105^c^ (0.056)
10–50-ppb arsenic	–	−0.158 (0.150)	–	−0.057 (0.056)
10–50 ppb*drink safe	–	0.140 (0.150)	–	−0.027 (0.053)
*R* ^2^	0.3294	0.3296	0.0489	0.0491
# observations	4511	4511	13,556	13,556

Samples: youths in the 2006 MICS with well contaminations from the 1998/2000 NHS

^a^Statistically significant at 0.01 level

^b^Statistically significant at 0.05 level

^c^Statistically significant at 0.10 level

Our first regression examined how groundwater arsenic affected expected years of education for males between 19 and 21 years of age. The dependent variable was the years of education attained by 2006. Table 4, column 1 contains estimated coefficients for the dummy variable “unsafe” and for the interaction of “unsafe” with the “drinks safely” dummy (with their robust, clustered standard errors in parentheses). Males living in the unsafe sub-districts are estimated to suffer a loss of one-half of a year of education relative to males who live in the safe sub-districts; males in the unsafe-water sub-districts who drink safely are estimated to experience no such deficit.

Table 5 reports estimates of the covariates’ coefficients from the regression reported in column 1 of Table 4. Parental education, parental wealth, and the presence of other youths in the household were all statistically significant with the expected signs. The local mean wealth variable had a statistically significant negative estimated coefficient.

**Table 5:** Regression 1 and 3 covariates’ estimated effects

	Years of education			Days attended		
	(19–21-year-olds)			(6–10-year-olds)		
Variable	Coef.	Robust std. err.	*t*	Coef.	Robust std. err.	*t*
Household wealth	1.366^a^	0.080	17.18	0.115^a^	0.026	4.41
Household wealth sqd.	−.194^a^	0.032	−6.12	−.031^a^	0.011	−2.75
Number of other youths in hours	−.130^a^	0.024	−5.39	0.001	0.011	0.11
Age	0.099^b^	0.052	1.91	0.055^a^	0.009	6.22
Head primary incomplete	0.247^b^	0.129	1.92	0.098^c^	0.043	2.29
Head primary complete	0.867^a^	0.121	7.17	0.160^a^	0.042	3.84
Head some secondary	1.486^a^	0.126	11.83	0.109^a^	0.042	2.60
Head secondary or more	2.425^a^	0.140	17.31	0.268^a^	0.046	5.87
Head non-std. schooling	−0.527	0.794	−0.66	0.376^a^	0.126	2.99
Head education missing	0.024	0.838	0.03	0.657^a^	0.126	5.23
Heard of arsenic prob.	0.557^a^	0.118	4.75	0.073^b^	0.038	1.92
Mean subdistrict wealth	−.812^a^	0.260	−3.12	−0.154	0.107	−1.44
Std. dev. sub-district wealth	0.202	0.333	0.61	0.018	0.139	0.13
Flood prone	0.224^b^	0.135	1.66	−.118^c^	0.055	−2.13
Garbage pile	−0.059	0.258	−0.23	0.125	0.211	0.60
Landslide prone	−0.570	0.679	−0.84	0.027	0.331	0.08
Industrial pollution	1.359^a^	0.324	4.20	−0.201	0.325	−0.62
Safe from eviction	0.443^c^	0.198	2.23	−0.001	0.055	−0.01
Squatter household	−1.062	0.666	−1.60	−0.092	0.153	−0.60

^a^Statistically significant at 0.01 level

^b^Statistically significant at 0.10 level

^c^Statistically significant at 0.05 level

Nearly two thirds of the sampled Bangladeshi youths faced wells with average arsenic exceeding the stricter WHO standard of 10 ppb. Table 4, column 2 reports coefficient estimates for a regression which added a dummy variable indicating an average local arsenic level between 10 and 50 ppb and an interaction between that dummy and the dummy for drinking safely.

Primary school boys’ behavior exposed the roots of older boys’ reduced schooling due to arsenic. Columns 3 and 4 of Table 4 report regressions with the explanatory variables used for columns 1 and 2, but with the dependent variable “days of school attended in the past week.” In Bangladesh’s 40-week school year, we estimated that boys who drank from unsafe water sources annually missed 4.8 to 6.9 more school days than did peers who drank safely. The estimated effect of arsenic levels between 10 and 50 ppb was imprecisely measured and statistically insignificant.

Table 5 reports estimates of the covariates’ coefficients from the regression reported in column 3 of Table 4. Parental education and parental wealth were statistically significant with the expected signs. The estimated effect of other youths in the household was small and statistically insignificant. The mean and standard deviation of local wealth were jointly significant. The environmental and housing variables were jointly insignificant, though being in a flood-prone area did have a statistically significant negative effect.

## Discussion

We employed data from Bangladesh’s 1998–2000 National Hydrological Survey and 2006 Multiple Indicator Cluster Survey to estimate the effect of groundwater arsenic on males’ education attainment and school attendance in Bangladesh. The former survey provided a large and geographically diverse sample of tube-wells whose arsenic contamination was known. The latter survey provided measures of education attainment, school attendance, and myriad control variables, including households’ responses to the question “Do you drink from arsenic free sources?” We concluded from our regression analyses of groundwater arsenic’s effects on education that, on average, drinking water unsafe by Bangladesh’s standards reduces by half a year, on average, a Bangladeshi boys’ accumulation of schooling and reduces by 5 to 7 days a year a young Bangladeshi boy’s school attendance. Hence, public health measures to shift drinking from unsafe to safe wells not only advance good health but also increase education attainment.

The estimated effect of drinking from Bangladesh-safe but WHO-unsafe wells is negative and of non-trivial magnitude but is quite imprecisely estimated. We are not alone in imprecisely estimating arsenic’s effects at low levels. The National Research Council reports that the shape of arsenic’s dose response curve for cancer remains unclear for low doses [49].

The regressions also offered a measure of our success in identifying the effects of arsenic: the estimates resolved the conundrum of Table 3 in which education attainment for the youths who reported drinking safely in the unsafe sub-districts was greater than the education attainment of the youths who drank safely by dint of living in a safe sub-district. In our regressions, which controlled for other determinants of education, we estimated that the sampled individuals who lived where groundwater was safe attained the same levels of education as sampled individuals who drank safely despite living where groundwater was unsafe. Passing this test lends increased creditability to the estimates of how much less education was attained by the individuals who drank from locally unsafe wells.

The results in Table 5 indicate that parental wealth, education, and awareness of the arsenic problem positively influence youths’ educational outcomes. Young males in wealthier sub-districts tend to attain less education than young males from other districts (when parental wealth is controlled for separately), but local wealth does not affect the school attendance by young boys. We interpret the local wealth variable as indicating the local income opportunities that compete with school for older youths’ time. Such opportunities are less apt to matter for young boys because for them, attending school is mandatory.

In unreported regressions, we found that adding males as young as 15 to the sample hardly changed arsenic’s estimated effects. Apparently, arsenic poisoning takes its education toll by age 15. Adding individuals as old as 25 cuts the estimated adverse effect to about three tenths of a year (but still confirms the finding of no adverse effect for those who drink safely). We attribute the lower estimate when including older individuals to older individuals having been less exposed to arsenic in the early 1980s than younger individuals were subsequently.

### Limitations

Correlational models like ours do not offer the iron-clad protection from bias that well-designed experiments can: in correlational models, omitted relevant variables can bias the results of an analysis. Our analysis, like most regression analyses, requires attention to such biases because we do not have as rich an array of covariates available to us as we would wish. In particular, our data are a single cross-section, not a panel, of individuals. Consequently, we cannot track the dynamic determinants of education attainment. Our reliance on a cross-sectional correlational model is limited with respect to three classes of variables: economic, health, and policy variables. Here, we attend briefly to the nature of the biases we risk by not having better measures of such variables.

To fully understand why an individual attains the schooling he or she does, one would favor a detailed examination of the individual’s economic circumstances over the course of the individual’s childhood. With only a single cross-section, we miss the fluctuations in households’ economic circumstances that affect education attainment. We observe a household’s wealth at a single moment of time, which provides only a partial picture of a household’s economic history. Because wealth fluctuates less over time than does income, observing a household’s wealth at one moment of time is more informative about the household’s economic circumstances over time than is observing the household’s current income. But wealth does, nonetheless, vary over time, and to the extent that wealth varied differentially across sub-districts with high and low levels of groundwater arsenic, our measures of arsenic’s effects on education attainment are biased. However, such biases are apt to be lessened by our model’s inclusion of both local aggregate mean wealth and local aggregate variation in wealth.

A potentially more serious concern is our lack of data about the non-arsenic related health status of individuals both over time and in the period we observe. If high levels of arsenic in groundwater are correlated with other health threats, such as malaria-carrying mosquitos, for which we have no measures, then our estimate of groundwater arsenic’s effect on education attainment will be biased. However, to the extent that individuals in a threatened area cannot avoid a specific health threat, both the estimated effect of arsenic contamination for those who drink arsenic-contaminated water and the estimated effect for those in the same sub-district who drink from a safe source would be biased toward reduced education attainment. Thus, if such health threats are substantially correlated with groundwater arsenic contamination, we would expect to see an effect of groundwater arsenic on education attainment for those who live in arsenic-unsafe sub-districts yet drink safely. This was not the case. A remaining concern for our results are health threats that are avoidable, as we would expect that households which avoided unsafe water would also have taken measures like bed nets to avoid diseases such as malaria. The question, then, is, “How correlated was arsenic contamination with such avoidable health threats?” We employ as control indicators of industrial pollution and garbage dumps in the vicinity of an individual’s home, but these are crude measures, so both avoidable environmental threats and avoidable ecological threats to health could cause biases in our results.

The third class of variables for which time series data would be valuable is policy-related variables. The effect of groundwater arsenic on residents of a sub-district is influenced by government policy. Moreover, government policy interventions are almost surely more intense in areas with the worst arsenic contamination. Both the extent of government policies in place during the childhoods of our observed individuals and the time path of those policies matter for the severity of groundwater arsenic’s effects. By focusing on 19- to 21-year-olds born between 1984 and 1986, we capture the effects of groundwater arsenic averaged across the policy practices in place between 1984 and 2006. Our reading of the empirical literature about arsenic policies’ efficacy suggests that policies shifted relatively few households from contaminated to safe supplies for two thirds of that time or more [33].

Our reliance on a single cross-section risks yet another bias. An arsenic level measured at one moment in time likely mismeasures individuals’ long-run exposure to arsenic, which is the truly relevant exposure. Consequently, our estimates suffer some attenuation bias. Since arsenic levels in wells do not change much over time, attenuation bias arises chiefly from individuals not always having lived in the sub-district in which they were observed in 2005. The more individuals moved between childhood and 2005, the greater their contribution to such attenuation bias. To reduce this bias, we restricted the sample of 19–21-year-olds to individuals who still lived with their parent or grandparent when sampled; this shrank the 19–21-year-old sub-sample by 17 %.

### The striking safety of drinking safely

Our multiple regression analysis controls for a large number of covariates that have been found by others to affect education attainment, including household wealth, parental education, number of other youths in the household, age, local environmental and economic indicators, and district of residence, as well as the household head’s awareness of the arsenic problem. Those controls negate many potential sources of bias. Our results also offer a striking check on the validity of our results. We estimate that males who did not report drinking from an arsenic-free water source and lived where tube-wells were unsafe obtained half-a-year less education than males who lived where tube wells were safe, but we also estimate that individuals who lived in unsafe sub-districts but drank water from arsenic-free sources suffered essentially zero adverse education effect from the local tube wells’ contamination. That the estimated effect of living in an unsafe sub-district disappears for those who did not drink the contaminated water strongly suggests that what we estimate as arsenic’s effect on education attainment was, indeed, arsenic’s effect and not a spurious result stemming from omitted variables.

## Conclusions

In 2012–2013, 20 million Bangladeshis regularly drank water containing more than 50 ppb of arsenic, the Bangladesh standard for contamination, and 40 million regularly drank water containing more than the 10 ppb of arsenic deemed unsafe by the World Health Organization [29]. Given the horrific health consequences of regularly drinking arsenic-contaminated water [1, 9, 10], Bangladeshis’ extensive reliance on arsenic-contaminated water, while much improved over levels of reliance in the past, still has severe adverse public health consequences. This paper’s analysis finds further that young Bangladeshi males who live where tube wells are unsafe and who do not report drinking from an arsenic-free source attain, on average, a half-year less education (among 19- to 21-year-olds) and attend school, on average, five to seven fewer days a year (among 6- to 10-year-olds) compared to other Bangladeshi males of those ages. Hence, Bangladeshi public health measures to shift drinking from unsafe to safe wells not only advance good health but also increase education attainment for males.

## Footnotes

1 The figures in Table 1 for 2000 are the percentages of wells above specified thresholds [27], while those for 2009 and those for 2012–2013 are based on tests of households’ drinking water [8, 30]. Since even in 2000 some households avoided sources known to be contaminated, the figures in Table 1 overstate how much households’ exposure has changed since 2000, but there is widespread agreement that households substantially reduced their reliance on arsenic-contaminated sources over the decade.

2 The regression results for females are available from the authors upon request. In our largest samples, girls aged 11–25, the estimated effect of drinking from contaminated sources is a tenth of a year decrease in education attained; that estimate has a *p* value of 0.102.
